# Two human milk–like synthetic bacterial communities displayed contrasted impacts on barrier and immune responses in an intestinal quadricellular model

**DOI:** 10.1093/ismeco/ycad019

**Published:** 2024-01-12

**Authors:** Charles Le Bras, Lucie Rault, Nolwenn Jacquet, Nathalie Daniel, Victoria Chuat, Florence Valence, Amandine Bellanger, Latifa Bousarghin, Sophie Blat, Yves Le Loir, Isabelle Le Huërou-Luron, Sergine Even

**Affiliations:** STLO, INRAE, Institut Agro, Rennes, 35042, France; Institut NuMeCan, INRAE, INSERM, Univ Rennes, Rennes-Saint Gilles, 35590, France; STLO, INRAE, Institut Agro, Rennes, 35042, France; STLO, INRAE, Institut Agro, Rennes, 35042, France; STLO, INRAE, Institut Agro, Rennes, 35042, France; STLO, INRAE, Institut Agro, Rennes, 35042, France; STLO, INRAE, Institut Agro, Rennes, 35042, France; Pediatric Department, CHU Rennes, CIC-Inserm 1414, Rennes, 35000 France; Institut NuMeCan, INRAE, INSERM, Univ Rennes, Rennes-Saint Gilles, 35590, France; Institut NuMeCan, INRAE, INSERM, Univ Rennes, Rennes-Saint Gilles, 35590, France; STLO, INRAE, Institut Agro, Rennes, 35042, France; Institut NuMeCan, INRAE, INSERM, Univ Rennes, Rennes-Saint Gilles, 35590, France; STLO, INRAE, Institut Agro, Rennes, 35042, France

**Keywords:** human milk, milk microbiota, immune system, intestinal epithelial barrier, synthetic bacterial community, gut homeostasis

## Abstract

The human milk (HM) microbiota, a highly diverse microbial ecosystem, is thought to contribute to the health benefits associated with breast-feeding, notably through its impact on infant gut microbiota. Our objective was to further explore the role of HM bacteria on gut homeostasis through a “disassembly/reassembly” strategy. HM strains covering the diversity of HM cultivable microbiota were first characterized individually and then assembled in synthetic bacterial communities (SynComs) using two human cellular models, peripheral blood mononuclear cells and a quadricellular model mimicking intestinal epithelium. Selected HM bacteria displayed a large range of immunomodulatory properties and had variable effects on epithelial barrier, allowing their classification in functional groups. This multispecies characterization of HM bacteria showed no clear association between taxonomy and HM bacteria impacts on epithelial immune and barrier functions, revealing the entirety and complexity of HM bacteria potential. More importantly, the assembly of HM strains into two SynComs of similar taxonomic composition but with strains exhibiting distinct individual properties, resulted in contrasting impacts on the epithelium. These impacts of SynComs partially diverged from the predicted ones based on individual bacteria. Overall, our results indicate that the functional properties of the HM bacterial community rather than the taxonomic composition itself could play a crucial role in intestinal homeostasis of infants.

## Introduction

Human milk (HM) promotes optimal growth and development of infants [1, 2]. Its positive impacts on the maturation of the intestinal immune system and barrier function partly account for its health benefits [3, 4]. HM contains numerous bioactive substances including proteins, lipids, and oligosaccharides that act as prebiotic for the infant gut microbiota [5, 6]. HM is also a major source of bacteria for infants with 10^5^ to 10^7^ bacteria being ingested daily by breast-fed infants [7]. HM microbiota was shown to contribute to the infant gut microbiota [8], with 5%–33% of the gut bacteria in infants under 6 months coming from the mother’s milk [9-11]. A direct impact of HM bacteria on the infant intestinal epithelium has started being explored as well. The potential of HM-derived *Bifidobacterium* and *Lactobacillus* strains to modulate the immune system and the epithelial barrier components has been well described using different cell models [12–17]. However, less is known about the role of all the other HM bacterial taxa on the intestinal functions.

HM microbiota is characterized by a low bacterial load, 10^3^–10^4^ colony-forming units (CFU)/ml, but a high diversity with dozens of genera and more than 200 species identified so far [18]. Bacteria present in HM originate from the maternal skin and gut microbiota as well as from the infant oral cavity [19, 20]. The latter origin is due to a retrograde flow back into the mammary duct during suckling, thus allowing an exchange of microorganisms between the infant mouth and the breast. Evidences show that HM composition is influenced by maternal (lactation stage, diet) and environmental (lifestyle, geographical localization) factors. The health impact on infants is still unclear [21]. The core HM microbiota includes *Bacillota* (formerly named *Firmicutes*), such as *Streptococcus*, *Staphylococcus*, and *Lactobacillus*; *Actinomycetota* (formerly named *Actinobacteria*), such as *Cutibacterium*, *Corynebacterium*, and *Bifidobacterium*; as well as *Pseudomonadota* (formerly named *Proteobacteria*) including *Serratia*, *Pseudomonas*, *Ralstonia*, and *Acinetobacter* [18, 22, 23]. *Staphylococcus* and S*treptococcus* are the most prevalent genera [22, 23]. HM also hosts a lot of anaerobic bacteria, as revealed by metagenomic approaches. The role of this great HM bacterial diversity on the infant gut homeostasis, apart from its role on microbiota, has been poorly addressed so far. A still unanswered question is whether HM microbiota, depending on its composition in terms of taxonomy and/or functionality, could display different impacts on the infant gut epithelium immune and barrier functions. To address this question, the role of HM bacteria on the intestinal barrier and immune responses was explored through a “disassembly/reassembly” strategy. HM strains covering the diversity of HM cultivable microbiota were first characterized individually and then assembled in synthetic bacterial communities (SynComs) using two human cellular models: peripheral blood mononuclear cells (PBMC) and a quadricellular intestinal model.

## Materials and methods

### Human milk sample collection and bacterial strain isolation and growth

The protocol of HM collection was approved by the Institutional Review Board of Poitiers Hospital (n°20.05.27.67526). Fresh HM was collected from 28 healthy (no infection and no antibiotic treatments), exclusively breast-feeding mothers between 2 and 6 weeks post-delivery (Supplementary Methods).

For the subsequent isolation of strains, aliquots of 100 μl of HM were plated on nine different selective and non-selective media to obtain the largest variety of bacteria (Table S1). A maximum of five colonies per medium, each with different morphologies, were selected, purified, and cultured in the corresponding liquid media. The isolates from blood agar medium were cultured on Brain Heart Infusion medium supplemented with 1% yeast extract (BHI-YEc) (Table S1). This led to a maximum of 50 strains per donor. Isolates were maintained at −80°C using the same media supplemented with 15% (w/v) of glycerol. Identification of the isolates was achieved by 16S rRNA gene sequencing as described in Supplementary Methods [24]. The 16 rRNA sequences of the isolates used in this study are available on the Data.Gouv.fr repository using the following DOI: https://doi.org/10.57745/EGIS5E.

Eighty-four isolates were selected to cover HM taxonomic diversity and limit the risk of redundancy (one isolate per species per mother) (Table S2). The 84 isolates were cultured (Tables S1 and S2), washed twice with Hanks’ Balanced Salt Solution, and suspended at different concentrations either in complete Roswell Park Memorial Institute (RPMI) medium or in Dulbecco’s Modified Eagle Medium without antibiotics nor fetal calf serum, for cell–bacteria interaction studies. Bacterial counts were estimated by OD_600 nm_ and expressed as CFU/ml. Bacterial population was determined in the growth conditions defined in Table S2, using the micromethod [25].

### Peripheral blood mononuclear cell and quadricellular intestinal model stimulation

About 5 × 10^5^ viable PBMCs (STEMCELL Technologies, Cambridge, USA; details in Supplementary Methods) were incubated in a 48-well tissue culture–treated plate with each of the 84 strains and 2 control strains (Table S2). PBMCs from two donors were stimulated with a multiplicity of infection (MOI) of 1:1 or 10:1 bacteria per cell for 24 h in complete RPMI (Supplementary Methods) at 37°C in a 5% CO_2_ water-saturated atmosphere (one replicate for each of the four conditions). After centrifugation (8000 *g*, 5 min, 4°C), supernatants were stored at −20°C until cytokine analysis by ELISA assay.

A quadricellular model was adapted from the tricellular model of Vernay *et al.* [26] and included in the apical compartment, human colon carcinoma 2 cell line (Caco2; enterocytes), human colorectal adenocarcinoma, methotrexate-resistant (10 mM) cell line (HT29-MTX-E12; goblet cells), and M cells: differentiated Caco2 cells with the action of the human Burkitt’s lymphoma B-cell line RAJI. M cells are involved in the translocation of antigens and bacteria from the gut lumen to the lamina propria. In addition, a human leukaemia monocytic cell line (THP1, immune cells) differentiated in macrophages was added in the basal compartment (Fig. S1, Supplementary Methods). This model was stimulated by each bacteria in the apical compartment at MOI 25:1 (three independent experiments in duplicate, *n* = 6). Preliminary experiments were carried out at MOI 1, 25, 50, 100, and 500:1 with four strains: Bb1, Ca2, Se4, and Lj. The MOI 25:1 was chosen for the experiment, as it induced a cell response without any deleterious effects on the cells. Alternatively, the model was stimulated by two SynComs of 11 bacterial strains each (four independent experiments in triplicate, *n* = 12). All the strains used for the assembly of the 2 SynComs (19 strains as 3 strains were common) have been registered in the collection of the CIRM-BIA Biological Resource Center (Rennes, France) or CIRM-BP Biological Resource Center (Tours, France) (https://www6.inrae.fr/cirm_eng/BRC-collection-and-catalogue; accessions number in Table S2). The 11 strains were added in equal proportion at a total MOI of 25:1, which means that each of the 11 strains in the SynCom was added at an MOI of 2.3 bacteria per epithelial cell (25/11 = 2.3). In addition to the negative control (quadricellular model without bacterial stimulation), an additional control (Control P) was used for the experiments with SynComs. It was composed of genera commonly used as probiotics: three *Bifidobacterium* (Bbi, Bb1, and Bb2) and two *Lactobacillus* (Lj and Lg) strains. Control P was introduced to mimic probiotic supplementation in some infant formulas, generally limited to few strains belonging to these genera. After 3 h of bacterial stimulation without antibiotic, 100 μg/ml gentamycin (Sigma-Aldrich, St. Quentin Fallavier, France) was added to the apical and basal compartments before an additional 21-h incubation. Antibiotic was necessary to avoid the proliferation of certain strains and subsequent medium acidification, which can adversely affect cell viability and epithelium integrity. A compromise was found by combining a 3-h incubation with live (metabolically active) bacteria and a 21-h additional incubation with antibiotic-treated and potentially lysed bacteria. Furthermore, the addition of antibiotic helped to maintain the proportions between bacteria within the SynComs and prevent an overrepresentation of fast-growing aerobic strains. After 24 h, media from the apical and basal compartments were collected and centrifuged (8000 *g*, 5 min, 4°C) and supernatants were stored at −20°C until cytokine analysis by ELISA assay.

### Analysis of immune and barrier functions (bacterial translocation, epithelial barrier integrity, cytokine production, and gene expression)

Individual bacterial translocation was measured on the quadricellular model at 3 h, just prior to the addition of gentamycin, by measuring the cultivable bacterial population in the basal compartment, using the growth media and conditions defined in Table S2. Results are expressed as the relative population in the basal compartment compared with the total population added in the apical compartment (Table S5). The epithelial barrier integrity was evaluated by measuring the transepithelial electrical resistance (TEER) of the epithelial cell layer at 0, 3, and 24 h of culture with a Millicell-ERS Voltmeter-Ohmmeter (Merck).

For cytokine production, interleukin (IL)-10 and tumour necrosis factor alpha (TNF-α) concentrations were measured using IL-10 and TNF-α ELISA kits (BD Biosciences, Franklin Lakes, NJ) according to the manufacturer instructions. For the experiment with PBMC, cytokine production was normalized by the median production of all bacterial stimulations for each donor to account for the variations of cytokine production between the donors. Results were expressed as the mean normalized IL-10 and TNF-α production for the two donors and the two MOI used (MOI 1:1 and MOI 10:1) ± standard error of the mean (SEM).

Extraction of total RNA of Caco2, HT29-MTX-E12, and M cells from one hand, and THP1 cells from the other hand, was performed as previously described [27]. Gene expression analysis was performed with the SmartChip Real-Time PCR System technology and with the 384-well plate PCR system for genes with detectable, but low, expression using the SmartChip system (cycle threshold (Ct) >28) (Table S3).

### Statistical analysis

Statistical analyses were performed using R (v 4.2.3) [28, 29]. Multidimensional scaling (MDS) was used for grouping bacteria and creating groups with different profiles. The number of groups was determined by combining the MDS with a K-means clustering analysis. The K-means clustering was tested with 3 to 6 groups, and the most stable clustering on 20 iterations was obtained with 5 and 3 groups for the PBMC and quadricellular models, respectively. For PBMC, eight variables were used: normalized IL-10 and TNF-α production for the two donors and the two MOI. For the quadricellular model, 41 variables were used: IL-10 in the apical and basal compartments, TNF-α in the basal compartment (TNF-α production in the apical compartment was included in the background of the method and not retained for statistical analyses), TEER and translocation measure, and the expression of 35 genes. Regarding the quadricellular model, an integrative approach, based on sparse partial least squares regression-discriminant analysis (sPLS-DA), was used to identify variables that discriminated MDS-defined groups of individual strains or the two SynComs. One-way analysis of variance (ANOVA) (PBMC; treatment as factor) and two-way ANOVA (quadricellular model; treatment and plate number as factors) followed by Tukey test were performed to evaluate the effect of groups of individual bacteria or SynComs on cells. *P*-values <.05 were considered significant.

## Results

### A representative collection of human milk bacteria

A collection of 1245 bacterial isolates (26 genus and 59 species) was constituted from 28 healthy breast-feeding mothers. All isolates belonged to four phyla, with a large majority of *Actinomycetota* and *Bacillota* (Fig. S2 and Table S4), three *Pseudomonadota*, and one *Bacteroidota*. Four genera were dominant, namely *Staphylococcus*, *Cutibacterium*, *Streptococcus*, and *Corynebacterium*, with prevalence of 100%, 93%, 86%, and 68% in HM samples, respectively.

### Human milk bacteria displayed a great diversity of immunomodulatory profiles on peripheral blood mononuclear cells

Eighty-four strains (Table S2) covering HM taxonomic diversity were evaluated for their immunomodulatory properties through their ability to induce IL-10 and TNF-α secretion in PBMC. Two strains were used as controls, namely *Propionibacterium freudenreichii* CIRM-BIA129 and *Lactococcus lactis* MG1363, that led, as expected, to high and intermediate IL-10 production, respectively [30, 31], thus validating the two PBMC donors (Table S2).

**Figure 1:**
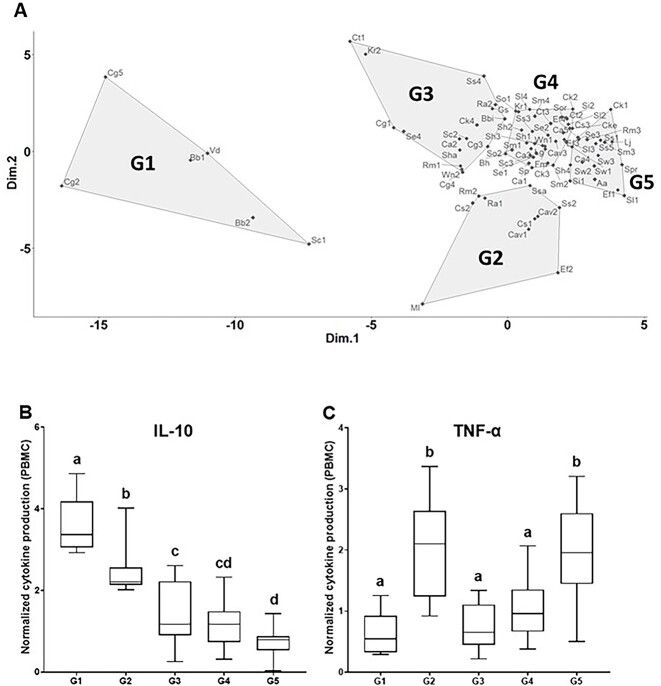
Distribution of 84 HM strains into five groups according to IL-10 and TNF-α secretion by PBMC stimulated by each strain; (A) classification by multidimensional scaling (MDS) of 84 HM strains into five groups, from G1 to G5 according to IL-10 and TNF-α secretion by PBMC stimulated by each strain; the full name of each strain and cytokine production is presented in Table S2; IL-10 (B) and TNF-α (C) secretion by PBMC stimulated by the different HM strains, organized by groups, from G1 to G5; values are expressed as median with the first and third quartiles; ^a,b,c,d^ groups without a common letter differ significantly (*P* < .05).

The ability of HM strains to stimulate cytokine secretion was highly strain-dependent and ranged for normalized IL-10 production from 0.02 to 3.96 and for TNF-α from 0.29 to 6.67 (Table S2). An MDS analysis classified the 84 strains into five groups with specific functional signatures (G1 to G5, including 6, 9, 15, 36, and 18 strains, respectively; Fig. 1A). The immunomodulatory properties differed between groups, with G1 group inducing the highest IL-10 and the lowest TNF-α secretion by PBMC, corresponding to a dominant “anti-inflammatory” profile. Conversely, G5 group induced the lowest IL-10 and the highest TNF-α secretion, corresponding to a dominant “proinflammatory” profile (Fig. 1B and C). Strains of G2 group stimulated both IL-10 and TNF-α secretion and were considered as “stronger stimulators,” exhibiting both anti- and proinflammatory properties. Finally, strains of G3 and G4 groups induced lower IL-10 and TNF-α secretion (“lower stimulators”). Although strains could be separated into five groups (based on both IL-10 and TNF-α production), these groups remained somewhat heterogeneous, as illustrated by a high range of IL-10 and TNF-α secretion within each group and no clear threshold between groups for either IL-10 or TNF-α (Fig. 1). The taxonomic composition revealed that strains of G1, G2, and G3 groups mostly belonged to *Actinomycetota*, whereas G5 group was dominated by *Bacillota* and G4 group displayed a more balanced profile (Table S2). Overall, within each genus, no clear pattern was observed with strains distributed into the five groups, as observed for *Corynebacterium* and *Staphylococcus*. Nevertheless, a trend was observed for some species such as *Cutibacterium granulosum* and *Bifidobacterium breve*, which were rather anti-inflammatory (G1) or low stimulators (G3). Conversely, *Enterococcus faecalis* strains were mostly in the G2 (stronger stimulators) and G5 (proinflammatory) groups, and all *Staphylococcus warneri* strains belonged to the G5 group.

### Human milk bacteria had an impact on barrier function in addition to their immunomodulatory properties

**Figure 2:**
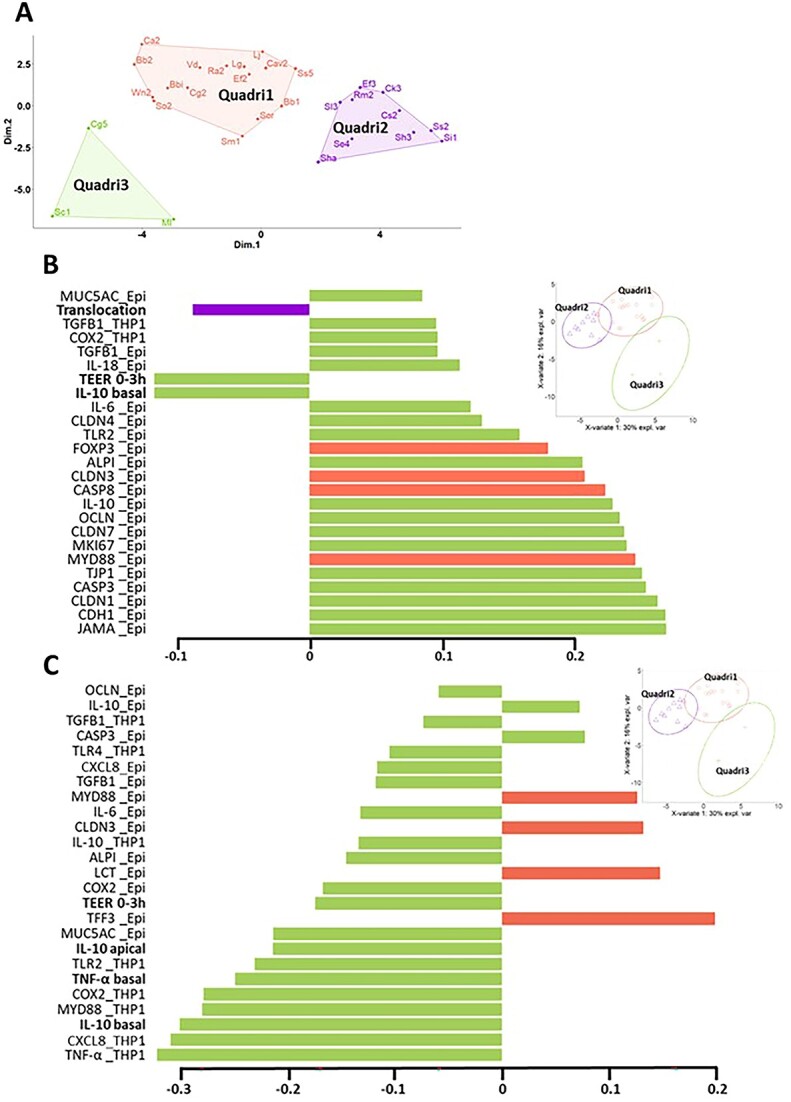
Distribution of 29 bacterial isolates into three groups according to their stimulation of cytokine secretion, their translocation, their impact on TEER, and on gene expression in the quadricellular model; (A) classification by MDS of the 29 selected strains into three groups, namely Quadri1, Quadri2, Quadri3, according to the 41 variables studied (IL-10 and TNF-α secretion, TEER changes, bacterial translocation and the expression of 27 and 8 genes by the epithelial cells and THP1, respectively) (see Tables S5 and S6 for the complete list of variables and values for each individual strain and for the groups of strains); list of the 25 most discriminating variables for Component 1 (B) and Component 2 (C) from the sPLS-DA analysis of the above-mentioned variables; variables of bacterial translocation, change in TEER between 0 and 3 h and IL-10 and TNF-α secretion, are indicated in bold; gene expression variables are in plain text; “_Epi” and “_THP1” indicate gene expression in epithelial cells (Caco2, HT29-MTX, and M cells) and THP1 cells, respectively; the corresponding protein names and functions are available in Table S6.

The impact on the intestinal immune, barrier, and apoptosis/proliferation functions of a subset of the strains characterized on PBMC was further explored using a quadricellular model of intestinal epithelium. The selection of these strains was based on the following criteria: several bacteria from each group (six from G1 and G2, five from G3, nine from G4, and three from G5) were selected to cover the immunomodulatory potential of HM bacteria, with a focus on groups G1 and G2 that displayed anti-inflammatory and high immunomodulatory properties, respectively. The second criterion that drove the selection of strains within each group was to keep the maximum of taxonomic diversity. Species belonging to HM prevalent genus were preferentially conserved, whereas species with both low prevalence and low PBMC-based immunomodulatory properties were discarded. Finally, 29 candidate strains belonging to 12 genera and 25 species were used for stimulation assay on the quadricellular model of epithelium (Tables S5 and S6). An overview of the HM bacteria impact on the quadricellular model was first achieved through an MDS analysis, using 41 variables for the 29 strains (Tables S5 and S6). The MDS analysis separated strains into three groups with a specific functional signature, namely Quadri1, Quadri2, and Quadri3 with 16, 10, and 3 strains, respectively (Fig. 2A). No major taxonomic biases were found between the three groups. Nevertheless, a balanced ratio between *Actinomycetota* and *Bacillota* was found in Quadri1 and Quadri3, whereas Quadri2 mostly included *Bacillota*. Besides, all *Bifidobacterium* and all *Cutibacterium* strains but one belonged to the Quadri1 group, while all *Staphylococcus* strains but one belonged to the Quadri2 group. On the contrary, other taxa such as *Streptococcus* strains were distributed in both Quadri1 and Quadri2 groups.

The functional properties of these three groups were then analysed through an integrative data analysis by sPLS-DA, allowing to identify discriminant variables between groups (Fig. 2B). The separation of Quadri1 and Quadri3 from Quadri2 groups was mostly achieved on Component 1, related to genes of the epithelial cells encoding tight junction proteins (TJP) (JAMA, CLDN1, TJP1, CLDN7, OCLN, CLDN3, and CLDN4), adherent junctions (CDH1), mucin (MUC5AC), marker of immune T cells (FOXP3), pattern recognition receptors and signal transduction mediators (MyD88, TLR2), and enzymes involved in cellular renewing (MKI67, CASP3, and CASP8) (Fig. 2C and Table S3 for detailed gene names). The separation of Quadri3 from the other two groups was mostly observed on Component 2 and was associated with IL-10 and TNF-α secretion, the corresponding gene expression, other immune-related genes in THP1 cells (IL-8, COX-2, and TGFB1), and in epithelial cells (IL-6 and IL-8), genes encoding pattern recognition receptors and signal transduction mediators (MyD88, TLR2) in THP1 cells (Fig. 2C).

**Figure 3:**
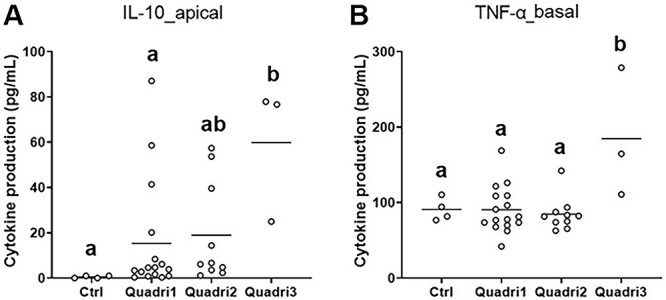
Impact of HM bacteria classified in three groups (see Fig. 2A) on IL-10 secretion in the apical compartment (A) and TNF-α secretion in the basal compartment (B) of the quadricellular model; “Ctrl” corresponds to the quadricellular model without any bacterial stimulation; each strain is represented by a circle and corresponds to a minimum of three replicates; the horizontal bar represents the mean of the group; ^a,b^ groups without a common letter differ significantly (*P* < .05).

The functional profiles of each group were further confirmed by ANOVA on each variable. Quadri3 strains exhibited higher immunomodulatory properties, as illustrated by a higher IL-10 (apical compartment) and/or TNF-α (basal compartment) secretion compared to the control and Quadri1 and Quadri2 strains (Fig. 3). These higher immunomodulatory properties of Quadri3 strains were also visible on the expression of immune, pattern recognition receptor, and signal transduction mediator genes in THP1 cells and epithelial cells. However, Quadri1 strains exhibited an intermediary immunomodulation profile in epithelial cells (Tables 1 and S6). Quadri2 strains hardly affected the expression of the immune genes in both epithelial cells and THP1 cells compared to the control. As noted for PBMC, although strains could be separated into three groups with different functional properties, these groups remained somewhat heterogeneous, as illustrated by a high range of IL-10 secretion within each group (Fig. 3, Table S5). The three groups also exhibited differential impacts on the barrier function with Quadri3 and, to a lesser extent, Quadri1, increasing the expression of genes implicated in tight junctions and mucin production compared to the control (Table 1). Conversely, Quadri2 strains decreased the expression of most of these genes as well as a gene encoding adherent junctions compared to control. Finally, regarding genes implicated in cellular renewing, the three groups decreased the expression of genes encoding caspase proteins (CASP3 and 8) compared to the control. Quadri2 strains exhibited the lowest expression of genes related to apoptosis and proliferation functions (Table S6).

**Table 1:** Normalized gene expression of the epithelial (Caco2, HT29-MTX, and M cells) and THP1 cells after 24-h stimulation of the quadricellular model by individual strains. Data are presented by groups of strains as determined by MDS analysis on the quadricellular model complete dataset (see Fig. 2A).^a^

Function	Gene name	Protein name	Control^b^	Quadri1	Quadri2	Quadri3	*P*-value
**Epithelial cells**							
Barrier	CLDN1	Claudin-1	1.37 ± 0.12^a^	1.32 ± 0.04^a^	0.91 ± 0.04^b^	1.48 ± 0.10^a^	<.001
	CLDN3	Claudin-3	1.18 ± 0.18^ab^	1.31 ± 0.04^a^	1.01 ± 0.06^b^	1.23 ± 0.06^ab^	.006
	CLDN4	Claudin-4	1.22 ± 0.12^a^	1.58 ± 0.06^a*^	1.38 ± 0.08^ab^	1.71 ± 0.18^b^	.024
	CLDN7	Claudin-7	1.24 ± 0.05^ab^	1.36 ± 0.04^ac^	1.08 ± 0.03^b^	1.58 ± 0.13^c^	<.001
	CDH1	Epithelial cadherin	1.36 ± 0.11^a^	1.36 ± 0.04^a^	0.91 ± 0.04^b^	1.49 ± 0.09^a^	<.001
	JAMA	F11 receptor	1.55 ± 0.13^a^	1.71 ± 0.05^ab^	1.18 ± 0.05^c^	2.01 ± 0.10^b^	<.001
	OCLN	Occludin	1.27 ± 0.02^ab^	1.51 ± 0.05^ac^	1.09 ± 0.05^b^	1.84 ± 0.20^c^	<.001
	TJP1	Tight junction protein 1 (ZO-1)	1.25 ± 0.14^a^	1.57 ± 0.05^b^	1.10 ± 0.05^a^	1.88 ± 0.14^b^	<.001
	MUC5AC	Mucin 5 AC	2.05 ± 0.54^a^	2.72 ± 0.19^a^	2.48 ± 0.37^a^	4.91 ± 0.93^b^	.005
	TFF3	Trefoil factor 3	1.11 ± 0.10^a^	0.74 ± 0.04^b^	0.65 ± 0.04^b^	0.52 ± 0.04^b^	<.001
Immune	IL-10	Interleukin 10	1.36 ± 0.20^a^	1.93 ± 0.14^b^	1.00 ± 0.09^a^	1.99 ± 0.08^b^	<.001
	TNF-α	Tumour necrosis factor	1.71 ± 0.25^a^	6.16 ± 0.68^b^	5.12 ± 0.95^ab^	7.93 ± 1.29^b^	.017
	IL-6	Interleukin-6	1.48 ± 0.48^a^	2.49 ± 0.22^ab^	1.86 ± 0.28^a^	4.01 ± 1.27^b^	.013
	TLR2	Toll-like receptor 2	1.18 ± 0.19^a^	1.28 ± 0.10^a*^	0.88 ± 0.12^a^	1.46 ± 0.12^a^	.049
	MYD88	Innate immune signal transduction adaptor	1.50 ± 0.13^a^	1.25 ± 0.05^a^	0.78 ± 0.06^b^	1.17 ± 0.08^a^	<.001
	COX-2 (PTGS2)	Cyclooxygenase-2	1.01 ± 0.14^a^	1.75 ± 0.09^ab^	1.89 ± 0.23^ab^	2.47 ± 0.53^b^	.012
	FoxP3	Forkhead Box P3	3.02 ± 1.84^a^	4.67 ± 0.59^a^	1.74 ± 0.27^b^	4.86 ± 2.40^ab^	.029
Apoptosis/proliferation	MKI67	Marker of proliferation Ki-67	1.39 ± 0.12^a^	1.21 ± 0.08^a^	0.67 ± 0.05^b^	1.33 ± 0.09^a^	<.001
	CASP3	Caspase-3	1.32 ± 0.06^a^	1.06 ± 0.03^b^	0.71 ± 0.05^c^	1.09 ± 0.10^ab^	<.001
	CASP8	Caspase-8	1.24 ± 0.08^a^	0.92 ± 0.04^b^	0.59 ± 0.06^c^	0.97 ± 0.13^ab^	<.001
Digestion	LCT	Lactase	3.89 ± 1.46^a^	1.71 ± 0.26^b^	1.47 ± 0.45^b^	0.42 ± 0.22^b^	.018
	ALPI	Alkaline phosphatase	1.61 ± 0.31^ab^	2.44 ± 0.16^a^	1.42 ± 0.17^b^	4.07 ± 0.62^c^	<.001
**THP1 cells**							
Immune	TNF-α	Tumour necrosis factor	1.09 ± 0.08^a^	1.21 ± 0.06^a^	1.24 ± 0.06^a^	2.27 ± 0.29^b^	<.001
	CXCL8	Interleukin-8	0.98 ± 0.15^a^	1.49 ± 0.15^a^	1.66 ± 0.19^a^	3.73 ± 0.77^b^	<.001
	COX-2 (PTGS2)	Cyclooxygenase-2	0.98 ± 0.20^a^	1.59 ± 0.16^a^	1.40 ± 0.15^a^	4.33 ± 1.18^b^	<.001
	TLR2	Toll-like receptor 2	0.95 ± 0.10^a^	0.98 ± 0.05^a^	1.12 ± 0.07^ab^	1.40 ± 0.20^b^	.035
	MYD88	Innate immune signal transduction adaptor	1.00 ± 0.09^a^	0.95 ± 0.03^a^	1.02 ± 0.04^a^	1.27 ± 0.06^b^	.008

a The quadricellular model of intestinal epithelium was individually stimulated by 29 HM strains for 24 h. MDS analysis clustered strains into three groups: Quadri1 (*n* = 16), Quadri2 (*n* = 10), and Quadri3 (*n* = 3) (Fig. 2A). Results of the mean epithelial cell gene expression of Quadri1, Quadri2, and Quadri3 groups are expressed as mean ± SEM. Differences between groups were assessed by one-way ANOVA followed by Tukey test. Different letters (a, b, c) for the mean gene expression of Quadri1, Quadri2, and Quadri3 groups indicate homogeneous statistical processing groups that were significantly different according to Tukey test. Only values of gene expression that statistically differed between groups (*P* < .05) are shown (see Table S5 for the complete dataset). * Indicates a tendency (*P* < .1) for each Quadri group to be different from control (Tukey test).

b “Control” was control cells without bacterial stimulation.

Altogether, these results revealed that Quadri3 strains and, to a lesser extent, Quadri1 strains displayed immunomodulatory properties on the epithelial and immune parts of the intestinal epithelium model and likely reinforced the epithelial barrier, while Quadri2 strains had poor immunomodulation properties and rather impaired the epithelial barrier.

### Assembly of human milk bacteria in two human milk-like synthetic bacterial communities that displayed contrasted impacts on the quadricellular model of intestinal epithelium

**Figure 4:**
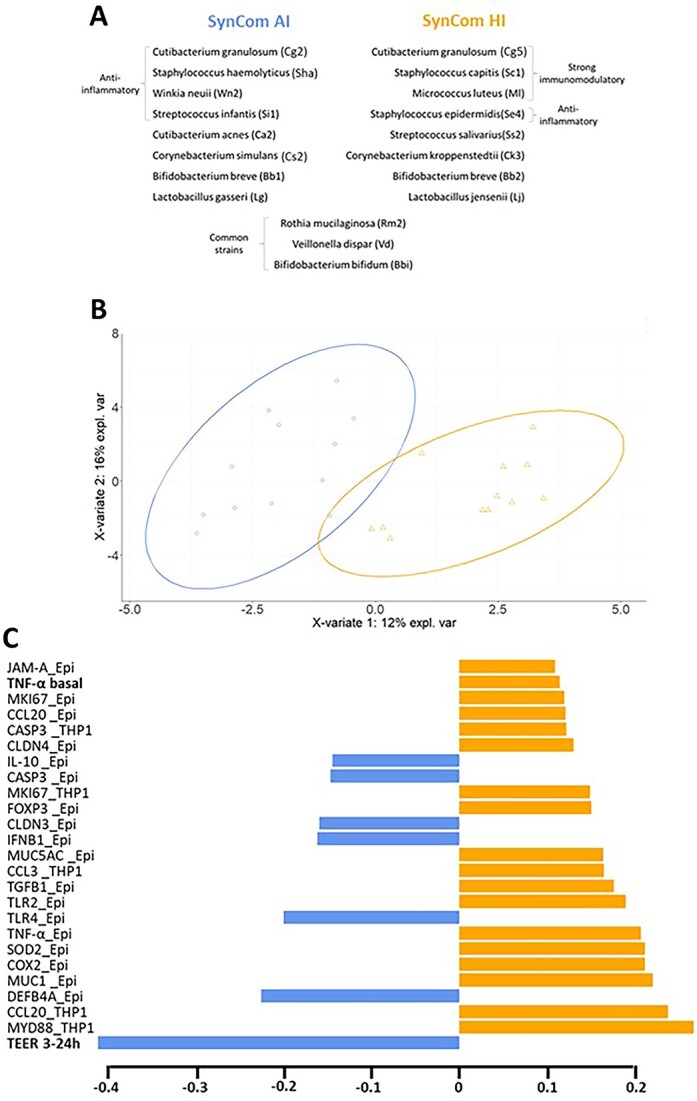
Functional profile of the two HM-like synthetic communities (SynComs); (A) composition of the two HM-like SynComs; the strains characterized with the quadricellular model (Fig. 3, Tables S5 and S6) were reassembled in two HM-like SynComs; (B) sparse partial least squares regression-discriminant analysis (sPLS-DA) of the two SynComs (*n* = 12 replicates) for the 50 variables: IL-10 and TNF-α secretion, TEER changes, translocation and the expression of 30 and 15 genes by the epithelial cells and THP1, respectively (see Tables S7 and S8 for the complete list of variables and values for each consortium and control); (C) list of the 25 most discriminating variables for Component 1 from the sPLS-DA analysis of the above-mentioned variables; variables of TNF-α secretion and change in TEER between 0 and 3 h are indicated in bold; gene expression variables are in plain text. “_Epi” and “_THP1” indicate gene expression in epithelial cells (Caco2, HT29-MTX, and M cells) and THP1 cells, respectively; the corresponding protein names and functions are available in Table S8.

To go further and better understand the role of HM bacteria that are normally present in HM as a complex bacterial community, the strains characterized with the quadricellular model were reassembled in two HM-like SynComs of 11 strains each, added in equal proportion. Eight strains were specific to each SynCom, and three strains were in common. The two SynComs were designed first to include the most prevalent HM genera and mimic the HM microbiota, albeit imperfectly. In each consortium, at least one representative of the four most prevalent genera of HM microbiota (*Staphylococcus*, *Cutibacterium*, *Streptococcus*, and *Corynebacterium*) was present, as well as representatives of other genera frequently found in HM such as *Bifidobacterium*, *Lactobacillus*, *Rothia*, and *Veillonella* (15). Second, due to the high variability in the immunomodulatory capacities of HM bacteria, strains were assembled to obtain two communities with theoretical contrasted immunomodulatory effects, based on their individual properties and notably their ability to stimulate the production of IL-10 and/or TNF-α by the multicellular model and PBMC (Fig. 4A). The SynCom AI (anti-inflammatory) was designed to display mainly anti-inflammatory properties and the SynCom HI (high-immunomodulatory) to display both anti- and proinflammatory properties. Theoretical values for each SynCom were calculated. Theoretical replicates were generated for each SynCom by averaging individual values of the 11 strains constituting the SynCom (each strain was individually tested in triplicate) (Tables S7 and S8). The theoretical values confirmed that SynCom AI should be mostly anti-inflammatory, whereas SynCom HI should stimulate both pro- and anti-inflammatory responses.

**Figure 5:**
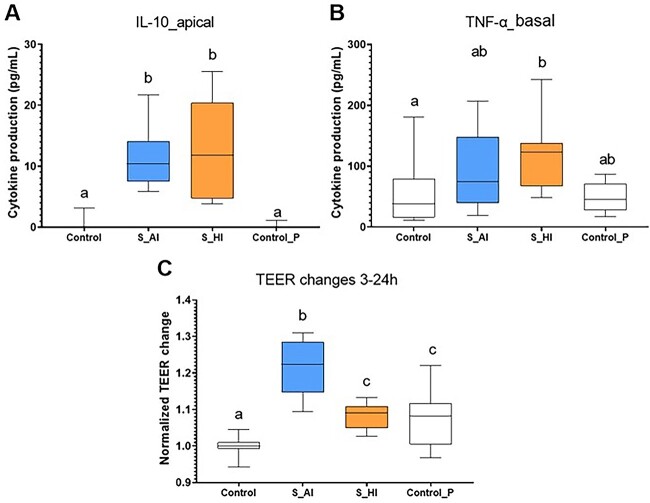
Impact of the two HM-like SynComs on IL-10 secretion in the apical compartment (A) and TNF-α secretion in the basal compartment (B) and TEER changes from 3 to 24 h of the quadricellular model (C); values are expressed as median with the first and third quartiles (*n* = 12 replicates for each SynCom); control, the quadricellular model without any bacterial stimulation (*n* = 10 replicates); Control P, an additional consortium of five strains including the three *Bifidobacterium* sp. and two *Lactobacillus* sp. strains from SynCom AI and/or SynCom HI (*n* = 7 replicates); ^a,b,c^ labelled medians without a common letter differ significantly (*P* < .05).

**Figure 6:**
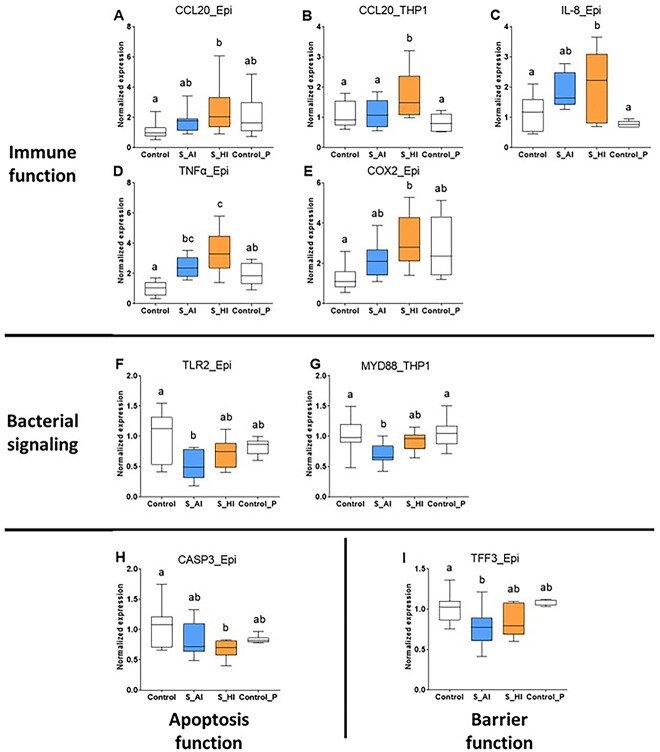
Impact of the two HM-like SynComs on the expression of genes of the quadricellular model; (A and B) CCL20, (C) IL-8, (D) TNF-α, (E) COX-2, (F) TLR2, (G) MYD88, (H) CASP3, (I) TFF3; “_Epi” and “_THP1” indicate gene expression in epithelial cells (Caco2, HT29-MTX, and M cells) and THP1 cells, respectively; the corresponding protein names and functions are available in Table S8; values are expressed as median with the first and third quartiles (*n* = 12 replicates for each consortium); “control” corresponds to the quadricellular model without any bacterial stimulation (*n* = 10 replicates) and “Control P” corresponds to an additional consortium of five strains including the three *Bifidobacterium* sp. and two *Lactobacillus* sp. strains from SynCom AI and/or HI (*n* = 7 replicates); ^a,b,c^ the lack of a common letter indicates that SynComs differ significantly (*P* < .05).

The functional properties of the two SynComs were characterized on the quadricellular model of intestinal epithelium. Using a PLS-DA analysis with 50 variables (Tables S7 and S8), the separation of the two SynComs was done exclusively on Component 1 (Fig. 4B and C). The 25 variables best discriminating the two SynComs included several variables related to immune functions, such as TNF-α secretion in the basal compartment, a majority of epithelial genes, and few THP1 genes related to immune functions and pattern recognition receptors or signal transduction mediators. Variables related to oxidative stress response, epithelial barrier (TEER variation between 3 and 24 h, genes encoding TJP, mucins, and cellular renewing) were also included. Functional profiles of each group were further refined by ANOVA on each variable (Figs 5 and 6, Tables S7 and S8). The two SynComs exhibited a similar anti-inflammatory impact, as illustrated by a higher IL-10 secretion in the apical compartment than the two controls (Fig. 5A). However, SynCom HI exhibited a higher global immunomodulatory profile compared to SynCom AI and the two controls, as illustrated by a higher TNF-α secretion (Fig. 5B) and an impact on the expression of different immune and oxidative stress–related genes (Fig. 6). Other differences included a decrease of the expression of genes involved in bacterial signalling by SynCom AI compared to the negative control, whereas SynCom HI had no significant impact (Fig. 6). Finally, regarding the barrier function, both SynComs increased the TEER between 3 and 24 h (Fig. 5C), more efficiently with SynCom AI, without a significant impact on the expression of genes encoding TJP or mucins (Table S8).

## Discussion

HM hosts a complex microbial community that receives increasing attention for its role in shaping the new born gut microbiota with potential short- and long-term health consequences. Several studies have reported the role of a few HM-isolated strains on the intestinal epithelial barrier and immune functions [15, 32, 33]. The present work aimed to further explore the role of HM bacteria as a complex community on the intestinal barrier and immune responses through a “disassembly/reassembly” strategy, and the combination of two *in vitro* models. This study highlighted that HM bacteria individually or assembled in SynComs can modulate the intestinal homeostasis with an impact on major immune and barrier functions. Data integration allowed the identification of several groups of strains with different functional signatures, these groups being loosely related to taxonomy. Finally, the assembly of HM strains in two SynComs with similar taxonomic composition but with strains that exhibited different properties individually resulted in contrasted impacts on the epithelium.

### Human milk bacteria display a great diversity in their potential to modulate intestinal epithelium immune and barrier functions

Using an untargeted approach (i.e. not focused on specific taxa), our study highlighted that HM bacteria display a broad range of effects on the intestinal epithelium immune system and barrier. The presence of immune- and barrier-modulating bacteria in HM has already been reported, especially for *Bifidobacterium* and *Lactobacillus* strains, with both pro- and anti-inflammatory profiles [12, 34] and generally a strengthening of the epithelial barrier with these taxa [15–17, 35–37]. Beyond *Bifidobacterium* and *Lactobacillus*, our results clearly point out the ability of the four prevalent and dominant cultivable genera of HM, namely *Cutibacterium*, *Staphylococcus*, *Corynebacterium*, and *Streptococcu*s in addition to other less prevalent and less dominant genera in HM, such as *Veillonella*, *Rothia*, or *Winkia*, to modulate the immune or barrier functions of the intestinal epithelium, at levels similar or even higher than those observed with *Bifidobacterium* and *Lactobacillus* strains. These genera have been poorly explored in the HM context, except for few *Streptococcus salivarius* strains isolated from HM that displayed immunomodulatory properties [38]. However, the above-mentioned species or genera have been shown to display immunomodulatory properties in other tissues, such as the skin for *Staphylococcus* and *Cutibacterium* [39–42], the mouth for *Streptococcus* [43, 44], or airways for *Veillonella dispar* (51). *Staphylococcus* can also impact the skin barrier functions by regulating TJP gene expression or host production of ceramides that helps to maintain skin integrity [45, 46].

One of the conclusions of this multispecies characterization of HM bacteria is the poor relationship between taxonomy and immune and barrier properties, as illustrated by the large taxonomic diversity within each group of strains displaying distinct functional properties on the PBMC or multicellular epithelium models. In addition, strains of a given genus, such as *Cutibacterium*, *Corynebacterium*, *Streptococcus*, and *Staphylococcus*, were generally distributed in different groups of strains. Analyses at the species level were inconclusive due to the low number of strains per species, but some trends seem to appear at the phylum level. G1 and G2 groups, that displayed anti-inflammatory properties on PBMC, were dominated by *Actinomycetota* and G5 group, exhibiting proinflammatory properties, was dominated by *Bacillota*. Moreover, Quadri2 group, that decreased TJP expression, was dominated by *Bacillota*. This apparent unbalanced distribution between *Bacillota* and *Actinomycetota* in the different groups should be evaluated on a larger set of strains. Apart from this apparent trend at the phylum level, the poor relationship between taxonomy and HM-bacteria properties suggests a strain dependence of their properties that has been widely reported and related to differences in terms of surface proteins, peptidoglycan, or exopolysaccharides [47, 48]. The low relationship between taxonomy and HM-bacteria properties is in agreement with the concept of functional redundancy that has been described for instance in gut microbiota and which implies that the functionality of the microbiota can be provided by taxonomically distant communities [49].

Interestingly, the intestinal quadricellular model revealed that Quadri3 and Quadri1 groups stimulated the immune functions and appeared to strengthen the epithelium barrier (increase of TJP expression), whereas Quadri2 group displayed poor immunomodulation properties and decreased TJP expression. This suggested that the stimulation of immune function and the expression of TJP genes were related, which was corroborated by a correlation analysis: a positive correlation was found between some TJP and IL-10 expression (correlation coefficient ρ = 0.7; *P*-value <.001) or IL-6 expression (ρ = 0.58; *P*-value <.001) in epithelial cells and to a lesser extent between the same TJP genes and TGFB1 and TNF-α expression in THP1 cells (ρ = 0.42; *P*-value = .03). This relationship between immune and barrier functions has already been observed by Le *et al*. who reported a TEER increase of a Caco2 cell layer in the first 30 h of interaction with activated T cells [50].

### Assembly of human milk bacteria into two synthetic bacterial communities: Beyond the sum of the effects of individual bacteria

Beyond investigating the potential of HM bacteria individually, the main goal of this study was to evaluate the impact of these strains in complex communities, mimicking the way they are assembled in HM. The immune and barrier functions of the intestinal epithelium were differently impacted according to HM strains assembled in SynComs. Of note, only a (very) low effect was observed with the “control P” SynCom, that only included *Lactobacillus* and *Bifidobacterium* strains, highlighting again a role of HM bacteria as a whole and the necessity to consider the diversity of HM bacteria.

A major question regarding the functionality of a bacterial community is whether it is the sum of individual properties. Interactions between bacteria such as mutualism or competition instead of neutralism may occur, as widely reported in microbial ecosystems [51–53]. In our study, the two SynComs exhibited properties close to the predicted ones, as revealed by the comparison between data obtained with SynComs and the theoretical values of these SynComs (i.e. an average of all the SynCom member effects). Stimulation of the immune system by both SynComs with a more proinflammatory profile of SynCom HI was expected, as illustrated on IL-10 production (Table S7) and the expression of COX-2 and TNF-α immune-related genes (Table S8). Conversely, for other variables, significant differences between SynComs and/or between SynComs and controls were not expected from the theoretical values, as for FOXP3 in epithelial cells, MYD88 in THP1 cells, or the TEER between 3 and 24 h (increased with SynComs AI and HI). These differences suggest that the final properties of the SynComs were not strictly the sum of effects of the strains composing it. The assembly of strains can confer new functionalities, as shown in plant studies where bacterial consortia increased photosynthetic and antioxidant systems compared to individual bacteria [54, 55]. Conversely, the assembly of strains may lead to a loss of functions. In probiotic clinical trials, the assembly of multiple strains was sometimes shown to be less efficient than single strains, with interactions between strains sometimes resulting in a loss of effect [56]. We observed such loss of effects with our SynComs that included strains that exhibited different and sometimes opposite functional properties, such as on TJP gene expression (Table S8). Nevertheless, the lower effects observed here with SynComs likely resulted from multiple opposite effects due to the interactions of each strain with the epithelium rather than from the lack of interactions, leading to a more balanced effect on the immune and barrier functions.

### From human milk synthetic bacterial communities to human milk microbiota

Altogether, our results highlight the potential of HM bacteria, beyond the well-characterized *Bifidobacterium* and *Lactobacillus* strains, to modulate the intestinal immune and barrier functions. This suggests that all these HM bacteria could play a role in the gut homeostasis of neonates and participate in the education of the immune system and the maturation of the intestinal epithelium barrier, in addition to their indirect contribution through their impact on the intestinal microbiota [8]. In agreement with this idea, maternal milk microbiota, as a major contributor of early gut colonization, was demonstrated to be involved in the establishment of intestinal immune and barrier functions in neonatal piglets [57]. *Streptococcus*, *Staphylococcus*, and *Bacillus* were specifically identified as transmitted bacterial taxa from sow milk to the intestine. Within HM microbiota, genera such as *Staphylococcus*, *Cutibacterium*, and *Corynebacterium* should be further evaluated for their contribution to gut homeostasis. These skin commensal bacteria have been proposed to play a role in the education of the immune skin system [40, 58–63] and could similarly participate in the education of the intestinal immune system.

The early life maturation of the intestinal immune system results in a balance between the anti-inflammatory response already present at birth and the acquisition of a complete proinflammatory response [64]. This balance allows the infant to tolerate commensal bacteria and food antigens, while, at the same time, providing optimal defence against pathogens. The presence of bacteria stimulating both anti- and proinflammatory responses in the HM microbiota would probably play a role in this maturation and contribute to the physiological inflammation peak necessary for the immune system maturation observed in breast-fed infants [65, 66]. Moreover, the differential impact of the two SynComs on the immune and barrier functions of the epithelium suggests that, depending on its composition, HM microbiota could affect the development of the infant intestinal immune system and barrier differently. Of note, the term “composition” refers rather to strain functionality than to taxonomic composition, as SynComs AI and HI exerted a different impact on the intestinal epithelium model, although they were quite similar in terms of taxonomy.

Although the use of SynComs has enabled us to highlight the potential role of the complex HM microbial community on the intestinal epithelium immune and barrier functions, one main limitation is that these simplified synthetic bacterial communities are still far from actual ecological communities. They were less diverse than HM microbiota and did not include several HM oxygen-sensitive species [22, 23, 67]. Nevertheless, considering the poor relationship between taxonomy and functionality, one can assume that these species would exert similar impacts on the intestinal epithelium. Besides, the SynComs included strains that did not originate from the same mother and that were assembled in equal proportions. Different proportions between strains, as usually observed in actual HM communities, may have a different impact on the intestinal epithelium with, e.g. an overrepresentation of the functionality of predominant taxa. Furthermore, the use of gentamycin in the multicellular model may have prevented some interactions between these bacteria and between bacteria and the epithelium, in contrast to the dynamics of interaction that may occur in real ecosystems. The use of actual ecological communities, isolated as a whole, from HM would undoubtedly be of great interest to confirm the results obtained with synthetic communities. This would be possible provided that the microorganisms are properly separated from all other milk components likely to interact with the epithelium, and that the cell layer integrity is preserved with a living and changing community. Another limitation of this study is the quadricellular model used, which is far from mimicking the complexity of the intestinal epithelium and especially the immune system, since only one immune cell type (macrophages) was used. At least macrophages are an important part of the immune cells in the lamina propria. Further understanding of the role of HM bacteria on infant gut homeostasis would also require consideration of the actual digestive environment, which can alter bacterial metabolism, viability, and integrity.

In conclusion, and despite the above-mentioned limitations, our study suggests that the functional properties of the HM bacterial community rely on a combination of strain-specific features rather than on the taxonomic composition itself. These functional properties could play a crucial role in intestinal homeostasis, including the intestinal epithelial barrier and immune system, in addition to the previously reported role on the gut microbiota. The disassembly/assembly strategy enabled us to demonstrate *in vitro* that the intestinal epithelium was differently impacted according to HM strains assembled in SynComs. Further investigations focussing on *in vivo* functional impacts of HM-based SynComs in infants would be useful.

## Supplementary Material

Supplementary_Figure_1_revised_ycad019

Supplementary_Figure_2_revised_ycad019

Supplementary_Table_1_revised_ycad019

Supplementary_Table_2_revised_ycad019

Supplementary_Table_3_revised_ycad019

Supplementary_Table_4_revised_ycad019

Supplementary_Table_5_revised_ycad019

Supplementary_Table_6_revised_ycad019

Supplementary_Table_7_revised_ycad019

Supplementary_Table_8_revised_ycad019

Supplementar_method_revised_ycad019

## Data Availability

All data generated or analysed during this study are included in this published article and its supplementary information files.
