# Supplementary material for: Two human milk–like synthetic bacterial communities displayed contrasted impacts on barrier and immune responses in an intestinal quadricellular model
Source: ISME Commun. 2024 Jan 12;4(1):ycad019. doi: 10.1093/ismeco/ycad019 (PMC10897888; doi:10.1093/ismeco/ycad019)
Supplement: Supplementary_Figure_1_revised_ycad019 [file supplementary_figure_1_revised_ycad019.docx]

**Supplementary Fig. S1:** Diagram of the set-up of the quadricellular model (see supplementary methods for more information).
