# Supplementary material for: Two human milk–like synthetic bacterial communities displayed contrasted impacts on barrier and immune responses in an intestinal quadricellular model
Source: ISME Commun. 2024 Jan 12;4(1):ycad019. doi: 10.1093/ismeco/ycad019 (PMC10897888; doi:10.1093/ismeco/ycad019)

**Supplementary Fig. S2:** Overview of the HM bacterial collection, through the prevalence and cumulated number of isolates of each genus. The phylum composition is also showed with the Bacillota in yellow, Actinomycetota in green, Pseudomonadota in orange and Bacteroidota in grey.


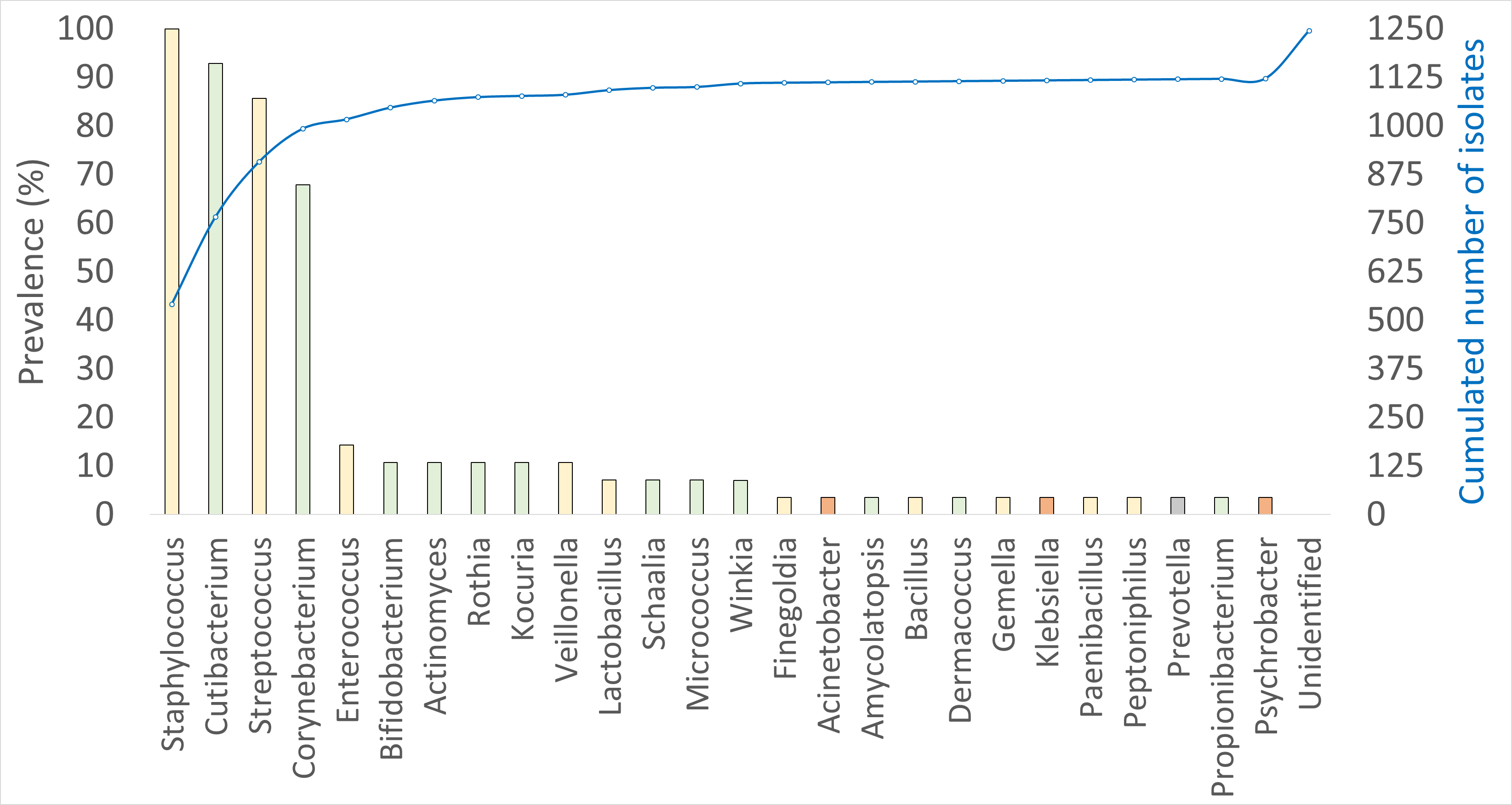

Supplement: Supplementary_Figure_2_revised_ycad019 [file supplementary_figure_2_revised_ycad019.docx]
