# Supplementary material for: Two human milk–like synthetic bacterial communities displayed contrasted impacts on barrier and immune responses in an intestinal quadricellular model
Source: ISME Commun. 2024 Jan 12;4(1):ycad019. doi: 10.1093/ismeco/ycad019 (PMC10897888; doi:10.1093/ismeco/ycad019)
Supplement: Supplementar_method_revised_ycad019 [file supplementar_method_revised_ycad019.docx]

**HM sample collection**

Forty-one healthy mothers who delivered vaginally at term a healthy baby and planned to exclusively breast-fed their infant, were recruited at the University Hospital Center of Rennes during the first week following delivery. Inclusion criteria were: vaginal delivery, baby at term, exclusive breastfeeding. Exclusion criteria were any formula feeding in addition to breast-feeding, sign of infection or drug administration (including antibiotics) to the baby and to the mother during the 3 months before delivery and the lactation period. 28 mothers meet the criteria at the time of sampling and their milk was collected between 2.0 and 6.0 weeks post-delivery.Before sampling, the breast was thoroughly washed with water and soap and rinsed with sterile physiological water (0.9% NaCl) before cleaning with Dakin^R^ (Sodium Hypochloride 0.5%) and individual sterile compress. Twenty mL of milk were then collected using a new and sterilized manual breast pump (Medela, Issy-les-Moulineaux, France). To minimize the exposure to O_2_, samples were stored in an anaerobic bag for a maximum of 18 h until use.

**Identification of the isolates**

Identification of the isolates was achieved by PCR amplification of the 16S rRNA gene directly on one representative colony, or following genomic DNA extraction. A representative colony was lysed for 10 min in 100 µl water at 95°C, followed by 5 min at 4°C. The lysate was centrifuged (10000 rpm, 5 minutes) and 4 µL supernatant was used in the PCR mix. Alternatively, DNA extraction was performed as follows: the bacterial suspension was lysed in 400-μL lysis buffer containing 20 mM TRIS-HCl (pH 8), 2 mM EDTA, 1% triton X100 and 0.4 g of 0.1 mM zirconium beads (VWR, Fontenay-sous-Bois, France) for 3x30s at 6800 rpm by using a Precellys Evolution device (Bertin Technology, Montigny-le-Bretonneux, France). Following incubation at 95°C for 7 min, samples were mixed for 15 s and centrifuged (18 000 g, 5 min, Room Temperature). Proteinase K treatment and DNA purification were performed using the Qiagen DNeasy tissue kit (Qiagen, Courtaboeuf, France), according to the manufacturer’s recommendations.

PCR amplification of the 16S rRNA gene was performed as previously described by Bouchard et al, 2015 (1) and sequencing of the PCR product was performed by LGC Genomics (Berlin, Germany). The forward and reverse sequences were assembled using Geneious R11.0.2 (Dotmatics, Boston, USA) and further compared to Genbank Database using BLAST. The highest score was retained for the identification. The identification was achieved at specie level when homology was higher than 99%. When homology search gave the same score with several species, isolates were identified at the genus level. The 84 isolates selected for the first characterization using the PBMC model were identified at species level with homology higher than 99.5%, except for 6 isolates (homology between 99 and 99.4%).

**Peripheral blood mononuclear cells**

PBMCs (#70025.3, Stemcell technologies, Cambridge, USA) from 2 different donors under 30 years of age and with a normal BMI were used. After thawing, PBMCs were washed with RPMI 1640 supplemented with 10% heat inactivated FCS and 1% penicillin-streptomycin (hereafter referred as to complete RPMI) and resuspended in complete RPMI.

**Intestinal cells and quadricellular model**

A quadricellular model was adapted from the tricellular model previously described by Vernay et al, 2020 (2). This model includes a tricellular model in the apical compartment formed by Caco2 (enterocytes), HT29-MTX-E12 (goblet cells) and M cells (located above the Peyer’s patch in the gut, involved in the translocation of antigens and bacteria from the gut lumen to the lamina propria where immune cells are located), and immune cells, THP1, in the basal compartment.

Intestinal epithelial Caco2 cells (obtained from Numecan in January 2021, originating from ATCC, ref ATCC HTB-37) and HT29-MTX-E12 cells (order from ECACC in January 2021, ref ECACC 12040401) were cultivated in DMEM High Glucose with L-glutamine supplemented with 10% heat inactivated Foetal Calf Serum (FCS; Biowest, Newry and Mourne, United Kingdom), 1% penicillin-streptomycin (Sigma-Aldrich, St. Quentin Fallavier, France) and 5% Non-Essential Amino Acid solution (Sigma-Aldrich, St. Quentin Fallavier, France) (hereafter referred as to complete DMEM). Lymphoblastoid RAJI cells (order from ECACC in February 2016, ref: ECACC 85011429) and monocyte THP1 cells (ATCC TIB-202) were cultivated in complete RPMI. Each cell line was grown at 37°C in a 5% CO2 water-saturated atmosphere in 25 or 75 cm^2^ flask (Corning Inc, Corning, NY, USA) until confluence.

After trypsin-EDTA treatment, Caco2 and HT29-MTX-E12 cells were seeded into the apical chamber of a Falcon® 24-well plate insert with a transparent 3 µm pore size PET membrane (Corning Inc, Corning, NY, USA) placed in a 24 well Falcon Support Companion Plate (Corning Inc, Corning, NY, USA). A total of 2x10^5^ cell/cm2 (i.e. 6.6x10^4^ cell/well) was added in 300 µL of complete DMEM with a ratio of 9 Caco2 cells for 1 HT29-MTX cell, corresponding to the ratio between enterocytes and goblet cells in the small intestine (3). 700 µL of complete DMEM was added to the basal compartment, medium was changed every 2 or 3 days. After 15 days of culture, 2.97x10^5^ RAJI cells, corresponding to a Raji/initial Caco2 cells ratio of 5:1, were added into the basal compartment to allow the differentiation of a part of Caco2 cells into a M cell phenotype. Vernay et al., evaluated the proportion of Caco-2 expressing characteristics of M cells at 7%, which is close to the M cell ratio in human Peyer’s patches which was reported to be less than 5% (4). Caco2, HT29-MTX and RAJI cells were co-cultured for 6 days with a daily change of half of the DMEM media after the first 3 days. At day 21, RAJI cells were discarded and co-cultured Caco2, HT29-MTX-E12 and M cells were washed 3 times with HBSS to remove all traces of antibiotics and FCS, before adding 300 µL of fresh DMEM medium without antibiotic and FCS. At day 20, in another 24 well Plate, 2x105 THP1 cells/well were cultured in 1 mL complete RPMI with 200 nM of Phorbol 12-myristate 13-acetate (PMA; Sigma-Aldrich, St. Quentin Fallavier, France) for 24 h, to allow differentiation and adhesion. Adhered THP1 were then washed 3 times with HBSS before adding 700 µL of DMEM medium without antibiotics and FCS. Then, the insert containing co-cultured Caco2, HT29-MTX-E12 and M cells was placed on the plate containing THP1 cells (see Fig S1 for the diagramm of the set-up of the model).

**References**

1. Bouchard DS, Seridan B, Saraoui T, Rault L, Germon P, Gonzalez-Moreno C, et al. Lactic Acid Bacteria Isolated from Bovine Mammary Microbiota: Potential Allies against Bovine Mastitis. PLOS ONE. 2015 Dec 29;10(12):e0144831.

2. Vernay T, Cannie I, Gaboriau F, Gall SDL, Tamanai-Shacoori Z, Burel A, et al. Bacteroides fragilis prevents Salmonella Heidelberg translocation in co-culture model mimicking intestinal epithelium. Benef Microbes. 2020 Aug 12;11(4):391–401.

3. Forstner J, Forstner G. Gastrointestinal Mucus. In: Physiology of the Gastrointestinal Tract 3rd ed. New York: Raven Press; 1994. p. 1255–84.

4. Giannasca PJ, Giannasca KT, Leichtner AM, Neutra MR. Human Intestinal M Cells Display the Sialyl Lewis A Antigen. Infect Immun. 1999 Feb;67(2):946–53.
